# Preparedness of Citizens in Al-Madinah City to Deliver Seizure First Aid Measures

**DOI:** 10.7759/cureus.49217

**Published:** 2023-11-22

**Authors:** Husain M Kateb, Omar Babateen, Husain B AlHabuobi, Sultan A Aljohani, Mohammed W ALjayyar, Taha I Khayat, Ahmad S Badawi

**Affiliations:** 1 Medicine and Surgery, Taibah University, Medina, SAU; 2 Department of Physiology, Umm Al-Qura University, Makkah, SAU

**Keywords:** epilepsy knowledge, public awareness, first aid, seizure, seizure first aid measures, cross sectional survey, general knowledge of seizures

## Abstract

Introduction

Epilepsy is a neurologic disease that causes a predisposition to recurrent seizure attacks. It affects a large number of people around the world and in Saudi Arabia. Seizures can be a source of distress for both the affected person and those witnessing them. Thus, being able to deliver first aid is important, as it improves safety and decreases the burden of visits to the emergency room that are unnecessary, since many attacks of seizure can be managed in the community. Weak knowledge regarding seizure first aid measures is reported many times across Saudi Arabia, with a high prevalence of misconceptions.

Methods

This study employed a descriptive cross-sectional design, utilizing a questionnaire-based approach. The data was collected from a sample of 1871 individuals residing in Al-Madinah City, Saudi Arabia. The participants completed a self-administered online questionnaire and ensured anonymity. The questionnaire used in this study was previously validated and used in another study. We used descriptive statistics to summarize the data, and Chi-square test was employed to establish the association between sociodemographic data and knowledge of seizure first aid.

Results

Most of the participants were females (70.0%, N=1310), and the majority (76.1%, N=1423) fell within the 18-30 age group. A high percentage were single (71.6%, N=1339), college graduates (64.3%, N=1201), and unemployed (58.7%, N=1099). The study results revealed that 31.9% (N=597) had a good knowledge level of epilepsy, while 68.1% (N=1274) had poor knowledge. Nearly half (48.4%, N=905) believed that epilepsy was caused by genetic factors, and 61.4% (N=1149) of the respondents reported loss of consciousness as the most common clinical symptom of epilepsy. With regards to correct action during seizures, 48.0% (N=899) knew to place the patient on their side, and 85.0% (N=1591) thought calling 997 ("the ambulance") was necessary if seizures lasted over five minutes.

The study showed a statistically significant association between the level of education, employment, and knowledge of epilepsy first aid management (p=0.001 and p=0.003, respectively). However, no significant associations were found between gender, age, marital status, and knowledge of epilepsy first aid management (p>0.005).

Conclusion

The study unveiled poor overall epilepsy knowledge among Al-Madinah City residents, with only 31.9% (N=597) demonstrating good knowledge. This indicates the community's limited ability to respond to seizures. Most citizens were unfamiliar with seizure first-aid, lacking the capability to provide assistance. A significant association was found between education, employment, and epilepsy first aid knowledge. Respondents with higher education had better epilepsy knowledge. Attending epilepsy education courses is vital for enhancing overall awareness and readiness to provide seizure first aid.

## Introduction

Epilepsy is a chronic noncommunicable neurologic disease characterized by recurrent seizures resulting from excessive electrical discharges [1]. The International League Against Epilepsy (ILAE) defines epilepsy as a disorder characterized by any of the following: two or more unprovoked seizures happening more than 24 hours apart, one unprovoked seizure with a risk of further seizures that is similar to the general recurrence risk after two unprovoked seizures, or a diagnosis of epilepsy syndrome [2]. Epilepsy affects millions worldwide and exerts a significant burden on healthcare and productivity. The condition has diverse causes, including birth-related brain damage, genetic factors, infections, strokes, and tumors, with approximately 50% of cases having an unknown underlying cause. Epilepsy exhibits a prevalence rate ranging from 4 to 10 per 1000 people, with an estimated 5 million new diagnoses annually [1]. In Saudi Arabia, the rate is approximately 6.54 per 1000 adults and children [3]. It disproportionately affects low- and middle-income countries due to factors like endemic conditions, limited medical infrastructure, and birth-related injuries. Beyond its medical and economic impact, epilepsy also poses social challenges, as the stigma and discrimination surrounding the condition often isolate individuals and discourage them from seeking proper treatment and support [1].

Witnessing a seizure can be distressing for both the individual affected and those present. It's crucial to understand seizure first aid to offer immediate help, ensuring safety. People with epilepsy might seek emergency care, potentially saving lives. However, many ER visits are unnecessary. Research shows that most ER visits involve known epilepsy cases with simple, community-manageable seizures, which are often influenced by factors exceeding clinical needs, leading to hospital admission even after achieving recovery. This, therefore, demonstrates the importance of knowledge about epilepsy, including reasons that necessitate epilepsy patients to visit the emergency department after experiencing a seizure [4].

Epilepsy incurs significant healthcare costs, with an average of $15,414 per patient in the USA [5] and an overall cost of €15.5B in the European Union in 2004, amounting to €2,000-11,500 per case [6]. Emergency care for epilepsy is costly and essential, but most ED visits involve diagnosed cases with uncomplicated, brief seizures. Hospital admissions are often needless. Managing such seizures in the community is feasible and should be promoted [7]. Seizure first aid is crucial, safeguarding individuals during seizures, preventing harm, and conserving ED resources. Those in contact with the public or people with epilepsy should possess seizure first aid knowledge due to the likelihood of encountering such situations [8]. Many global and local studies have assessed public knowledge of seizures, epilepsy, and first aid. In Grenada, a study of 200 adults uncovered misconceptions, including the belief that epilepsy is limited to childhood, that seizures are linked to low brain activity, that physical contact can induce epilepsy, or that it's contagious. These misconceptions heighten the risk of additional harm to individuals with seizures or epilepsy [9].

In an Iranian study of 342 primary school teachers, their knowledge and first aid measures for epilepsy were assessed. Results revealed that 91.8% (N=314) recognized epilepsy as a neurological condition, but some had misconceptions or lacked awareness. A troubling number exhibited potentially harmful responses during seizures, leading to insufficient knowledge [10]. In a local cross-sectional study in Al-Qunfudah, Saudi Arabia, involving 1,171 schoolteachers in 2022, most had moderate knowledge. However, 88.13% (N=1032) lacked first aid training, with 84.63% (N=990) never receiving seizure first aid training [11].

In the Eastern Province of Saudi Arabia, a study on teachers found that 90% (N=381) lacked seizure first aid training, and 61.5% (N=260) had poor epilepsy knowledge [12]. A nationwide cross-sectional study in Saudi Arabia indicated widespread insufficient knowledge about seizures and their management [13]. In the southwestern region of Saudi Arabia, a study of 1230 participants revealed superstitious beliefs, with 16.7% (N=205) attributing epilepsy to spirit possession and 14.1% (N=173) to the evil eye [14]. These local studies underscore the need for public education on seizures and epilepsy due to prevalent misconceptions [11-14].

This study aimed to assess the general knowledge of seizures among Al-Madinah City residents, determine their familiarity with essential seizure first aid measures, and evaluate their ability to administer first aid when necessary. These objectives provide insight into the community's preparedness and knowledge in managing seizure-related situations.

## Materials and methods

Study design and population

This study employed a questionnaire-based cross-sectional design that targeted the general population of Al-Madinah City that are older than 18 years of age. We conducted this study from August 2023 to November 2023. This design provides a general overview of the level of knowledge of the residents in Al-Madinah but cannot establish causality.

Sampling technique and sample size

The population of Al-Madinah City is estimated to be 1,570,000 [15]. Given this information, the sample size is determined to be 385 with a 95% confidence level and a 5% margin of error. The sample size was calculated using the Sample Size Calculator by Raosoft, Inc., which is available online. We utilized the convenience sampling technique for its high practicality in this type of research, as it helps spread the questionnaire to reach a wide range of individuals who reside in Al-Madinah. However, selection bias is a limitation for the use of this method of sampling.

Inclusion and exclusion criteria

In this study, we included individuals aged 18 years and older and those who live in Al-Madinah. People who were younger than 18 years of age and those who resided outside Al-Madinah were excluded from this study.

Data collection

We collected the data for this research using an online, self-administered survey, which was distributed on several platforms, including WhatsApp, Twitter, and Instagram. We collected the data from individuals who live in Al-Madinah, Saudi Arabia.

Data collection tool

A previously validated questionnaire, which was used in a previous study by Habbash et al. (2022), was employed. The questionnaire consisted of three main sections. The first section gathered participants' sociodemographic data, which serves as the independent variables of this study, including age, gender, educational level, and income. The second section assessed features related to epilepsy, including knowledge about the disease, its causes, and its management. The third section focused on assessing participants' perceptions of first aid, addressing first aid measures for seizures, and their knowledge about them. We used multiple-choice questions to evaluate knowledge regarding epilepsy and calculated a knowledge score based on the number of correct answers in this section, with a higher score indicating better knowledge. Similarly, for knowledge regarding perception of first aid, a knowledge score was calculated based on participants' responses to the questions. Subsequently, a comparison was made among various individual characteristics.

Data presentation and statistical analysis

Data were collected using a self-administered, previously validated online survey. Informed consent was obtained from all participants. The survey was distributed electronically using Google Forms. After gathering responses, they were checked for completeness, and incomplete surveys were excluded. The data was exported using Excel version 16.48 (Microsoft, Redmond, WA, USA). The information of the participants remained confidential. After that, the data were transferred to SPSS (IBM Corp., Armonk, NY, USA) for analysis. Continuous variables, such as age, were reported as mean ± SD. Categorical variables, like gender, were described as frequencies and percentages. A Chi-square test was used in the comparison of categorical variables, with a significance level set at p < 0.05.

Ethical consideration

The data gathered from the participants was kept confidential and only used for scientific research. Participation was entirely voluntary, and informed consent was put on the first survey page. Sensitive data, like names or identification numbers, were not collected, as they are not needed for analysis or publication. This study is consistent with the principles of the Declaration of Helsinki, and all participants were informed about the nature and objectives of the study at the start of the questionnaire. Potential conflicts of interest were identified prior to starting this research in order to maintain objectivity. Ethical approval to conduct this study was obtained from the Biomedical Research Ethics Committee of Umm Al-Qura University (approval number: HAPO-02-K-012-2023-09-1713).

## Results

A total of 1871 participants completed the questionnaire. The majority of the participants, 70.0% (N=1310), were female, with more than half of the participants, 76.1% (N=1423), belonging to the age group of 18-30 years. In terms of marital status, 71.6% (N=1339) were single, 26.7% (N=500) were married, 1.0% (N=18) were divorced, and 0.7% (N=14) were widowed. As for education level, the majority of the participants (64.3%, N=1201) were college graduates, 58.7% (N=1099) were unemployed, 15% (N=281) related to field jobs, and 12.6% (N=236) were students.

**Table 1:** Socio-demographic background of the participants (N=1871). Socio-demographic characteristics presented in frequencies (n) and proportion (%).

Socio-demographic data	Category	Frequency and Proportion n (%)
Gender	Male	561 (30.0%)
	Female	1310 (70.0%)
Age	18- 30 years	1423 (76.1%)
	31-49 years	348 (18.6%)
	Above 50 years	100 (5.3%)
Marital status	Single	1339 (71.6%)
	Married	500 (26.7%)
	Divorced	18 (1.0%)
	Widowed	14 (0.7%)
Education level	Primary school	5 (0.3%)
	Middle school	18 (0.7%)
	High school	529 (28.4%)
	Diploma	7 (0.4%)
	College	1201 (64.3%)
	Higher education	111 (5.9%)
Employment	Field job	281 (15.0%)
	Office job	218 (11.7%)
	Unemployed	1099 (58.7%)
	Student	236 (12.6%)
	Others	37 (2.0%)

**Figure 1:**
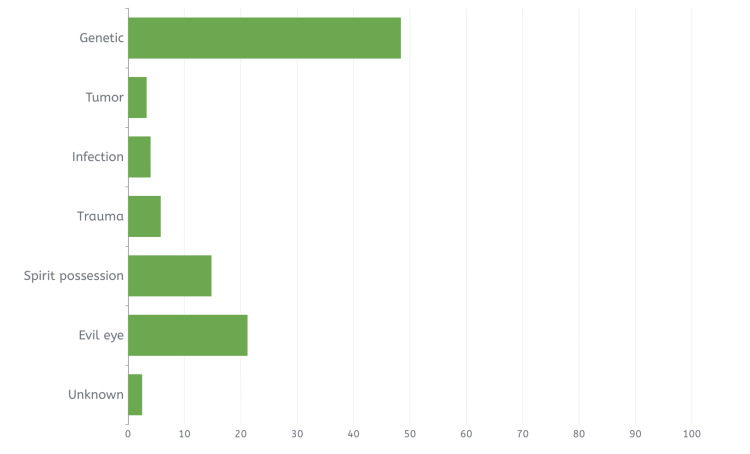
The bar chart shows the distribution of answers regarding the cause of epilepsy.

**Figure 2:**
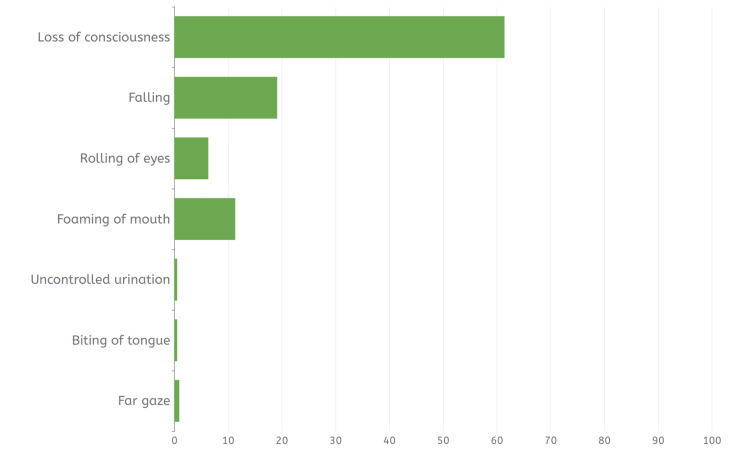
The bar chart shows the percentage of answers regarding the symptoms of epilepsy.

Table 2 below illustrates the epilepsy-related characteristics of participants. The findings revealed that the majority of the respondents (93.4%, N=1748) claim to have knowledge about epilepsy, with 37.1% (N=679) of them learning about the condition from the media. Regarding the cause of epilepsy, about half of the participants (48.4%, N=905) believed that epilepsy is caused by genetic factors, with trauma accounting for 5.8% (N=109), infection for 4% (N=75), and tumors for 3.3% (N=61). In contrast, 21.2% (N=397) of the respondents believed it was caused by evil eye, while 14.8% (N=277) cited spirit possession, as shown in Figure 1. As for their knowledge of the clinical symptoms of epilepsy, the majority (61.4%, N=1149) cited loss of consciousness, as demonstrated in Figure 2.

**Table 2:** Epilepsy-related characteristics of the participants. Epilepsy-related characteristics presented in frequencies (n) and proportion (%).

Epilepsy-related characteristics	Categories	Frequency and Proportion n (%)
Do you know what epilepsy is?	Yes	1748 (93.4%)
	No	123 (6.6%)
If the answer is yes, how did you learn about it?	Media	679 (37.1%)
	Relatives of friends	604 (34.6%)
	Seminars	80 (6.6%)
	Academic learning	138 (7.9%)
	Doctors	247 (14.1%)
Do you personally know someone with epilepsy?	Yes	651 (34.8%)
	no	1220 (65.2%)
What do you think the cause of epilepsy is?	Genetic	905 (48.4%)
	Tumor	61 (3.3%)
	Infection	75 (4.0%)
	Trauma	109 (5.8%)
	Spirit possession	277 (14.8%)
	Evil eye	397 (21.2%)
	Unknown	47 (2.5%)
What do you think are the symptoms of epilepsy?	Loss of consciousness	1149 (61.4%)
	Falling	358 (19.1%)
	Rolling of eyes	118 (6.3%)
	Foaming of mouth	211 (11.3%)
	Uncontrolled urination	10 (0.5%)
	Biting of tongue	10 (0.5%)
	Far gaze (strain)	15 (0.9%)
Choose the following statements that you think are true	All children with epilepsy have the same symptoms	317 (16.9%)
	Epilepsy is confined to children only	28 (1.5%)
	Epilepsy is a contagious disease	23 (1.2%)
	Epilepsy can be cured	405 (21.6%)
	None of the above is true	1098 (58.7%)
Do you think a child can have a seizure and not be recognized?	Yes	1402 (74.9%)
	No	469 (25.1%)
Do you think epilepsy is a lifetime disorder??	Yes	1030 (55.1%)
	No	841 (44.9%)
Do you know what to do if an epileptic child has an attack in front of you?	Yes	925 (49.4%)
	No	946 (50.6%)
What do you think is the most appropriate way to manage a child with epilepsy during the attack?	Do nothing and call his parents	192 (10.3%)
	Restrain the child	67 (3.6%)
	Put something in his mouth to prevent his tongue from swallowing	756 (40.4%)
	Keep him sitting or hold him upright	409 (21.9%)
	None of the above	447 (23.9%)

**Figure 3:**
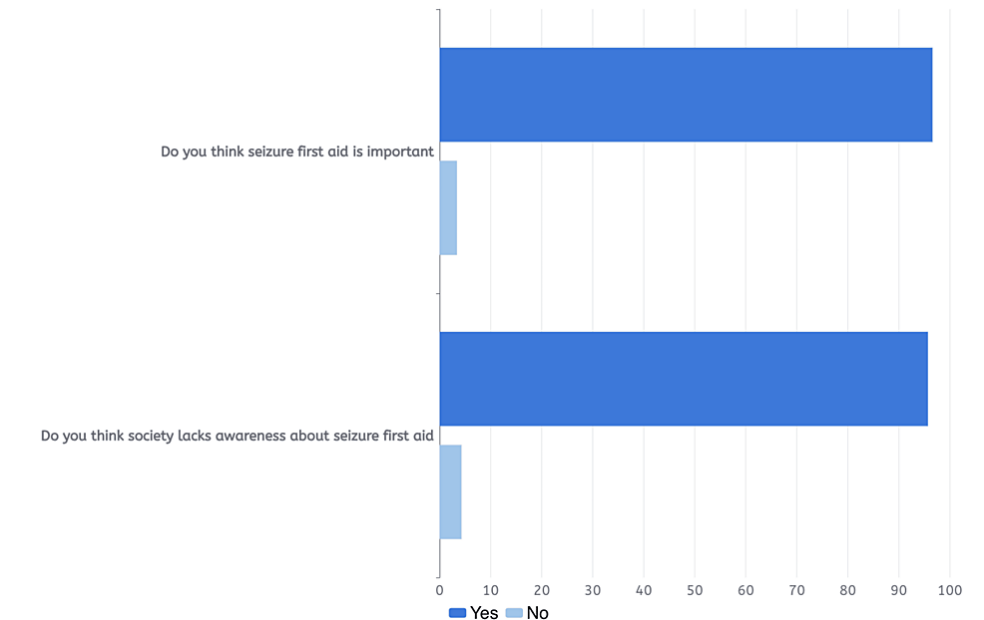
The bar chart compares the participants' responses to the importance of seizure first aid and whether society lacks awareness about it or not.

Table 3 below displays the first aid perception characteristics of the participants. A significant majority (96.6%, N=1807) believed in the importance of seizure first aid, with 95.7% (N=1791) expressing concerns about society's awareness of it, as shown in Figure 3. Over two-thirds (68.4%, N=1279) recognized that restraining a person during a seizure was incorrect, with 81.2% (N=1038) acknowledging the risk of injury to both the patient and themselves. About 48.0% (N=899) correctly understood that tilting the patient on their side was the appropriate action during seizures. After a seizure, only 5.9% (N=111) were aware of the importance of explaining the episode to the patient in simple terms. The majority (85.0%, N=1591) believed it necessary to call 997 (the ambulance) if a seizure lasted over five minutes.

**Table 3:** First aid perception-related characteristics First aid perception-related characteristics represented in frequencies (n) and proportion (%).

Questions	Answers	Frequency and Proportion n (%)
Do you think seizure first aid is important?	Yes	1807 (96.6%)
	No	64 (3.4%)
Do you think society lacks awareness about seizure first aid?	Yes	1791 (95.7%)
	No	80 (4.3%)
Do you think restraining the person during the attack is wrong behavior?	Yes	1279 (68.4%)
	No	592 (31.6%)
If the answer was “Yes”, why?	It may cause injury to me or themselves	1038 (81.2%)
	It’ll make the seizure get better	241 (18.8%)
How to prevent a person during a seizure from swallowing their tongue	Place a wallet or other	561 (30.0%)
	Put them on their side	899 (48.0%)
	Hold their head still	287 (15.4%)
	Do Nothing	124 (6.6%)
After a seizure has passed and the person is fully awake, what should you do?	Help the person sit in a safe place	1352 (72.3%)
	Tell them what happened in simple terms	111 (5.9%)
	Comfort the person and speak calmly	408 (21.8%)
Do you think calling 997 “the ambulance” is necessary?	Yes	1480 (79.1%)
	No	391 (20.9%)
When do you think you need to call 997 “the ambulance”?	If the seizure lasts more than 5 minutes	1591 (85.0%)
	If the person is injured	130 (6.9%)
	If the person has difficulty of breathing after the jerking stops	114 (6.1%)
	If it is the person’s first known seizure	36 (2.0%)

Table 4 below illustrates the relationship between age, gender, level of education, and occupation in terms of knowledge regarding first aid management of seizures. The results indicate a statistically significant association between level of education and knowledge of first aid management of epilepsy (p=0.001). Similarly, a statistically significant association was found between employment and knowledge of first aid management of epilepsy with (p=0.003). However, no statistically significant associations were observed between gender, age, marital status, and knowledge of first aid management of epilepsy (p>0.005). The study results show that 31.9% (N=597) had a good knowledge level of epilepsies, while 68.1% (N=1274) had poor knowledge.

**Table 4:** The association between gender, age, marital status, level of education, employment and knowledge of first aid management of epilepsy. Association between demographic variables and knowledge of first aid management of epilepsy represented as frequencies (n), proportion (%), and P-values. P-values were considered statistically significant at (p<0.05). *Statistically significant

		Knowledge of first aid management of epilepsy		
Variables	Category	Poor; n (%)	Good; n (%)	P-value
Gender	Male	1270 (67.9%)	601 (32.1%)	0.852
	Female	1218 (65.1%)	653 (34.9%)	
Age	18- 30 years	1235 (66.0%)	636 (34.0%)	0.321
	31-49 years	1233 (65.9%)	638 (34.1%)	
	Above 50 years	1431 (76.5%)	440 (23.5%)	
Marital status	Single	1190 (63.6%)	681 (36.4%)	0.259
	Married	1336 (71.4%)	535 (28.6%)	
	Divorced	1302 (69.6%)	569 (30.4%)	
	Widowed	1240 (66.3%)	631 (33.7%)	
Education level	Primary school	1276 (68.2%)	595 (31.8%)	0.001*
	Middle school	1259 (67.3%)	612 (32.7%)	
	High school	1304 (69.7%)	567 (30.3%)	
	Diploma	1255 (67.1%)	616 (32.9%)	
	College	1240 (66.3%)	631 (33.7%)	
	Higher education	1220 (65.2%)	651 (34.8%)	
Employment	Field job	1353 (72.3%)	518 (27.7%)	0.003*
	Office job	1377 (73.6%)	494 (26.4%)	
	Unemployed	1353 (72.3 %)	518 (27.7%)	
	Student	1037 (55.4%)	834 (47.6%)	
	Others	1418 (75.8%)	453 (24.2%)	

## Discussion

The study aimed to assess citizens' general seizure knowledge in Al-Madinah City, determine their familiarity with seizure first aid, and evaluate their ability to administer such aid. The sample predominantly comprised young adults, aged 18-30, with mostly single, college-educated, and unemployed individuals. The findings revealed that 93.4% (N=1748) of the respondents had knowledge about epilepsy. Among them, 37.1% (N=679) acquired this knowledge through media, while 34.6% (N=604) learned about it from relatives and friends. Furthermore, the study indicated that only 31.9% (N=597) of participants demonstrated adequate understanding and experience in dealing with epilepsy, its causes, and treatment. The estimated score clearly indicates the insufficient ability of the public to deal with a patient having a seizure in the community. Nevertheless, the low awareness levels in this study were unsurprising and align with previous research. The findings mirror those of the study conducted by Al-Dosary et al. [13] in Saudi Arabia, which found that only about one-third of the female teachers in primary schools in Riyadh expressed the ability to provide first aid to their pupils with epilepsy despite their exposure to mandatory training on first aid management in a school setting. Similarly, the study conducted by Alaqeel et al. [16] in Saudi Arabia found that the majority of male teachers in primary and intermediate schools in southern Saudi Arabia were not able to provide first aid to their epilepsy students during seizure attacks, although they had received mandatory government training on first aid management.

The study revealed a significant level of knowledge regarding the causes of epilepsy, with 61.5% (N=1150) of respondents correctly identifying genetic factors, tumors, infections, and trauma as contributing factors. These findings align with those reported in Al-Dossari et al.'s study [17], which found substantial awareness of epilepsy causes, including trauma, infection, and tumors, among Al-Kharj City residents in Saudi Arabia. However, 21.2% (N=397) of respondents believed in the evil eye as a cause, while 14.8% (N=277) attributed epilepsy to spirit possession, indicating limited understanding of the condition's nature and causes. This reflects improved public awareness of epilepsy causes compared to the study by Al-Dossari et al. in the Alkharj region in 2021, where 46.5% (N=186) believed in demonic possession or evil spirits as causes and more than half cited the evil eye. With regards to first aid, the majority (96.6%, N=1807) believed that seizure first aid was important. Forty-eight percent (N=899) of the respondents knew the correct action during seizure first aid should be to tilt the patient on their sides. Regarding the correct action during an epileptic fit, 85.0% (N=1591) of the respondents reported a need to call 997 “the ambulance” if the seizure lasted for more than five minutes. These results regarding the correct action during epileptic fit reflect a better awareness in comparison to the results of the study done by Alkhotani et al. [18] in Makkah, Saudi Arabia, which found that out of a sample of 426 teachers evaluating their knowledge of seizure first aid, the majority (55%, N=234) chose to open the patient’s mouth and insert an object; this indicates that the overall participants’ knowledge regarding epilepsy and its management was poor among study participants in the region.

The results established a statistically significant association between the level of education, employment, and knowledge of first aid management of epilepsy. However, there was no statistically significant association between gender, age, marital status, and knowledge of first aid management of epilepsy (p>0.005). The study revealed that the respondents with post-high school education qualifications had a higher average score of epilepsy knowledge than those with lower levels of education. Epilepsy educational campaigns and programs could enhance participants’ knowledge and, hence, the preparedness of citizens in Al-Madinah City to deliver seizure first-aid measures. The students in primary and high school should be encouraged to get a proper education and develop positive skills, which are important to prepare them for future epilepsy care in Saudi Arabia.

Study limitations

Since this study utilizes a cross-sectional design, it does not establish causality but only captures a snapshot in time. Moreover, the convenience sampling method can limit the representativeness of the sample and may introduce selection bias. Using an online questionnaire may also limit the representativeness of the sample, as it may exclude those with no internet access and those who don't use digital platforms. Social desirability bias is also a limitation of self-administered questionnaires, resulting from overreporting of knowledge. Furthermore, the data cannot be generalized to the entire population of Saudi Arabia, as it was conducted only in Al-Madinah City. Also, this study is not able to assess the depth of knowledge and the presence of confounding factors due to the limitations of the study design and data collection tool.

## Conclusions

The study revealed that most Al-Madinah City residents had inadequate knowledge of epilepsy and seizures. Only 31.9% (N=597) demonstrated good understanding of epilepsy, its causes, and management. This underscores the public's limited ability to assist someone experiencing a seizure in the community. Many citizens lacked familiarity with seizure first-aid measures and the capacity to provide such aid. A significant association existed between education, employment, and knowledge of epilepsy first aid. Respondents with post-high school qualifications exhibited better epilepsy knowledge. Attending epilepsy education courses is crucial for enhancing overall knowledge, awareness, and preparedness for delivering seizure first aid.
